# Targeting Mediators of Smoking Persistence with Intranasal Insulin

**DOI:** 10.3389/fphar.2017.00706

**Published:** 2017-10-04

**Authors:** Ajna Hamidovic

**Affiliations:** Pharmacy Practice, College of Pharmacy, University of Illinois at Chicago, Chicago, IL, United States

**Keywords:** intranasal insulin, smoking, weight loss, anhedonia, impulsivity

## Abstract

Rapid-acting, non-irritating nasal treatment options for smoking cessation pharmacotherapy are lacking. The halt in development is due, in part, to difficulty in delivering compounds across the blood brain barrier. Recently, in both human and animal models, insulin was shown to be capable of being transported to the cerebrospinal fluid and various brain regions via the “nose-to-brain” pathway, which bypasses the blood brain barrier, but is not free of its own unique, though different from blood brain barrier, challenges. This review will first evaluate and critique pharmacokinetic and pharmacodynamic evidence of intranasal insulin (i.e., nose-to-brain) delivery. As intranasal insulin has been shown in clinical trials to be effective in reducing nicotine cravings, in the remainder of the review, hypothesis-generating literature for additional mediators (i.e., other than the already shown nicotine craving) of smoking persistence will be reviewed. In particular, weight gain, impulsive behavior, and anhedonia have been shown to contribute to the inability to quit smoking. For each of these, after reviewing how the mediator promotes smoking, intranasal insulin literature from animal and clinical models will be critiqued in assessing whether a hypothesis may be generated that intranasal insulin may alleviate it, thereby potentially contributing to a successful smoking cessation outcome.

## Introduction

Smoking remains the single most preventable cause of morbidity and mortality (Wipfli and Samet, 2016), killing almost half a million people each year (U.S. Department of Health and Human Services, 2014). Of the estimated 40 million smokers in the United States (Centers for Disease Control and Prevention, 2011), only ~6% are successful in quitting smoking (Centers for Disease Control and Prevention, 2011). Therefore, development of novel and more efficacious smoking cessation treatments is critical.

**Figure 1:**
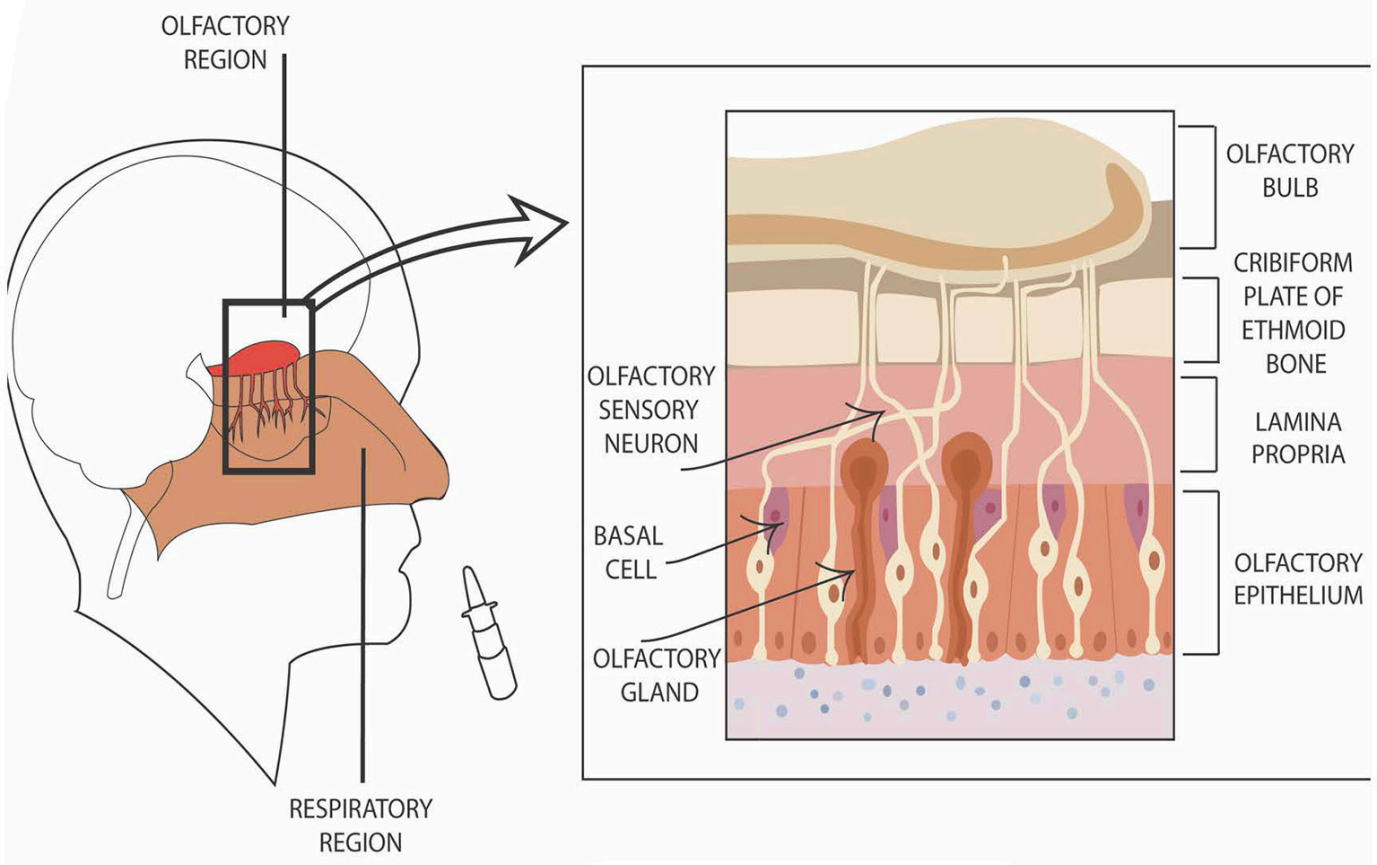
Graphic presentation of direct nose-to–brain entry. The left side of the figure shows the location of nasal olfactory region. The right side of the figure depicts the location of olfactory sensory neurons in reference to additional cell types in the region.

A significant barrier to tobacco use disorder drug development is the inability to deliver therapeutic compounds through the blood brain barrier (BBB). Whereas, <2% of small compounds effectively cross the BBB, larger molecules and peptides are nearly impenetrable (Pardridge, 2005). The intranasal route of drug delivery may overcome these difficulties. Direct “nose-to-brain” delivery is accomplished by a targeted deposition of compounds to the highly innervated, odor-detecting sensory system (Figure 1, left side). Combining immunofluorescence and immunoelectromicroscopy, Steinke et al. (2008) have demonstrated that the glia-like sustenular cells form tight junctions in order to constitute a barrier from the environment. Hence, the system is not free from drug delivery challenges. Nonetheless, the cellular properties of the barrier are different from those of the BBB, and may be a platform for delivering compounds which are BBB-impenetrable (reviewed in Warnken et al., 2016).

Earlier studies have shown that insulin can be delivered to the brain via the nose-to-brain pathway without significant systemic insulin absorption. This review will first evaluate the pharmacokinetic and pharmacodynamic evidence, following which it will provide the rationale for hypothesizing whether the neuropeptide may target significant mediators of smoking continuation.

## Intranasal insulin–pharmacokinetic and pharmacodynamic relevance

Compounds entering cerebrospinal fluid (CSF) through the olfactory epithelium in nasal cavity do so via olfactory sensory neurons (Ruigrok and de Lange, 2015) which innervate lamina propria (Figure 1, right side). They move along the nerves both intra- and extracellularly. The extracellular transport is fast (minutes to half an hour; Dhuria et al., 2010) and occurs as a result of combination of extracellular convection (i.e., bulk flow) and propagation of action potentials (Lochhead and Thorne, 2012). Intracellular mechanisms (endocytosis and passive diffusion transport) take hours to days. Numerous biologics, including insulin and other peptides, as well as proteins and gene vectors/stem cells have been delivered to the brain via direct nose-to-brain mechanism (for reviews, see Lochhead and Thorne, 2012; Kozlovskaya et al., 2014; Ruigrok and de Lange, 2015).

Insulin is not an unfamiliar peptide in the CNS. Challenging an earlier position that under normal physiological conditions insulin does not pass into the brain or CSF, a monumental radioimmunoassay study by Margolis and Altszuler (1967) found insulin in the CSF to be ~25% of plasma CFS. Insulin enters the brain via saturable, insulin receptor-mediated transcytosis (Havrankova et al., 1981; Duffy and Pardridge, 1987; Woods et al., 2003; Smith and Gumbleton, 2006; Meijer et al., 2016), and its elimination from the brain is either insulin-specific or non-specific as part of the overall CSF outflow. That is, insulin can be specifically degraded by insulin degrading enzyme in brain parenchyma and CSF (Behl et al., 2009). An alternate route of elimination is via general CSF outflow to lymphatic vasculature and venous sinuses (Louveau, 2015).

Therapeutic intranasal delivery of insulin reaches CSF in humans (Born et al., 2002) and specific brain regions in rodents (Salameh et al., 2015). In an attempt to deliver insulin to the CSF, but bypass the bloodstream and avoid insulin's potent hypoglycemic systemic effects, Born et al. (2002) administered 40 IU of insulin intranasally to 36 subjects. Serum and CSF samples were taken at −10, 0, 10, 20, 30, 40, 60, and 80 min. Compared to the placebo condition, insulin CSF levels were higher 30 and 40 min after intranasal administration. There were no significant serum insulin differences. Brain penetration of insulin in humans has not been studied; instead, indirect measurements of brain “energetics” following intranasal insulin administration have been conducted in two studies. In a randomized, placebo-controlled study, (Jauch-Chara et al., 2012) utilized magnetic resonance spectroscopy to measure changes in motor cortex ATP, which reflects intracellular energy content. Intranasal insulin (40 IU) resulted in increased ATP, without peripheral insulin changes. The second study showing insulin's effect on brain energetics was a randomized, placebo-controlled study, evaluating memory function of Alzheimer's disease patients after 4 months of treatment with intranasal insulin. Intranasal insulin improved memory in mild/moderate disease patients (Craft et al., 2012). A subset of patients (*n* = 40) in that study underwent positron emission tomography before and after treatment. Placebo-assigned participants showed progressive hypometabolism (i.e., reduced glucose metabolism)—a typical feature of Alzheimer's disease progression. The effect was observed in several brain regions of placebo-treated patients while intranasal insulin-treated patients showed a significant dampening of the hypometabolic progression.

Results of human studies show that intranasal insulin, independently of its peripheral effects, if any, produces a measurable central PK/PD result. In two clinical trials, we have recently shown that intranasal insulin, without circulating insulin changes, rapidly decreases nicotine cravings (Hamidovic et al., 2017). Though nicotine craving is central to tobacco use disorder (Tiffany and Wray, 2012), additional mediators—i.e., cessation-induced weight gain, anhedonia and increased impulsivity—also contribute to relapse. The remainder of this review specifies the role of these mediators in smoking continuation and evaluates whether there is evidence to support the hypothesis that insulin may target them.

## Weight gain

Weight gain of nine pounds on average (Tian et al., 2015) occurs in ~75% (Lycett et al., 2011) of abstinent smokers. Significant differences between regions exist, with weight gain being the highest in North America and lowest in Asia (Tian et al., 2015). As not all gained weight can be attributed to smoking cessation, adjustment for other variables, most notably age, shows a weight gain of about 11 pounds in the United States (Veldheer et al., 2015). Post-cessation weight gain considerably impacts health, but does not surpass the adversity of continuing to smoke (Siahpush et al., 2014). Nonetheless, smoking cessation increases the risk of developing type II diabetes and this risk is mediated by weight gain. In a prospective Atherosclerosis Risk in Communities Study (Yeh et al., 2010), the investigators found that the risk of diabetes for abstinent smokers in the first 3 years *increases* in comparison to the risk of smokers. The risk is similar to the risk of smokers years 4–10, and stabilizes to the same risk as a never smoker after ~12 years. The hazard ratio of diabetes among former smokers (quit > 3 years), new quitters (quit ≤ 3years), and continuing smokers were 1.22 (CI, 0.99–1.50), 1.73 (CI, 1.19–2.53), and 1.31 (CI, 1.04–1.65), respectively. The risks were substantially attenuated after an adjustment for weight gain.

Whether intervention strategies targeting body weight concerns or actual weight gain *per se* improve smoking cessation outcomes is not well understood. An early paper (Perkins et al., 2001) investigating this question found that participants in a cognitive behavioral treatment designed to address concerns about weight gain were more successful quitters than participants in the behavioral intervention group designed to attenuate the actual weight gain. However, since this publication, much has rapidly changed in the United States with an “epidemic” fraction of individuals being classified as obese. Future strong prospective studies paralleling ongoing changes in obesity rates can adequately address various components of this complex question.

Laboratory studies provide information on physiological and cellular mechanisms by which withdrawal from nicotine mediates weight gain observed in population studies. Nicotine withdrawal causes profound changes in the homeostatic and hedonic regulatory mechanisms, and weight gain occurs due to increase in caloric intake (Perkins et al., 1992; Filozof et al., 2004). Caloric intake is an important, but seemingly not the only contributor to weight gain (Munafò et al., 2009). However, the impact of non-dietary influences on weight gain is not clear. While some studies have shown a change in energy expenditure after acute (12 h) abstinence (Perkins et al., 1989), whether these changes are relevant to smoking cessation weight gain has been brought into question due to mixed findings (reviewed in Chiolero et al., 2008).

Although we are just starting to understand the mechanisms through which nicotine suppresses weight gain, the arcuate nucleus in the hypothalamus has emerged as a site of nicotine's effect. The nucleus contains two main neuronal networks—the anorexigenic pro-opiomelanocortin (POMC) and cocaine-amphetamine-regulated-transcript (CART) neurons as well as the orexigenic acting neuropeptide Y (NPY) and agoutirelated peptide (AgRP). The POMC neurons further synapse onto the second order neurons which contain the hippocampal melanocortin receptor 4 (MC4-R)—a seven-transmembrane G-protein coupled receptor. The resulting increase in the second order MC4-R signaling provides crucial inhibitory tone that restrains food intake. In fact, both nicotine (Mineur et al., 2011) and insulin (Seeley and Woods, 2003) activate pro-opiomelanocortin (POMC) neurons in the arcuate nucleus of the hypothalamus through α3 β4 and insulin receptors, respectively. However, elimination of insulin signaling in POMC neurons is not sufficient to produce any effect on weight. Whereas, global neuronal insulin receptor deletion results in an obese phenotype (Brüning et al., 2000), mice lacking insulin receptor only in POMC have unchanged food intake or energy expenditure (Chong et al., 2015). Instead, lack of insulin signaling in the mouse and fly specifically in the NPY neurons leads to an obese phenotype with dysregulated energy expenditure (Loh et al., 2017).

In addition to the above-described insulin's effect on the homeostatic regulation, an additional, hedonic locus of insulin's effect on food valuation was recently evaluated in a human pharmacologic fMRI study (Tiedemann et al., 2017) comparing healthy vs. diabetic participants. Whereas, mesolimbic connectivity between ventral tegmental area and nucleus accumbens—as well as subjective rating of food palatability—was reduced in the healthy control group receiving intranasal insulin (vs. placebo), the effect was not observed in the diabetic group. The interpretation of results, however, is complicated because the single blood sample—taken 50 min after spray administration—resulted in higher circulating insulin concentration in the healthy control group upon receiving insulin (vs. placebo) condition. As such, it is difficult to attribute the study finding specifically to the central effects of insulin. A related study (Jauch-Chara et al., 2012), though, measured insulin, glucose, and c-peptide blood levels every 10 min through a 100-min time period after spray (intranasal insulin or placebo) administration. Though there were no differences in the three measures between the intranasal insulin and placebo conditions, the intranasal insulin group had a markedly reduced total caloric intake. This study, coupled with animal studies showing insulin's reducing effects on food intake arising from the hypothalamic (Loh et al., 2017) and mesolimbic (Labouebe et al., 2013) loci, provide the foundation for an evaluation of intranasal insulin as a weight management agent during the course of smoking cessation. If shown to be effective, this would not only reduce the incidence of type II diabetes in abstinent smokers, but may also increase smoking cessation rates by reducing weight gain concerns, thereby having a substantial impact on public health.

## Impulsivity

Much progress has been made recently in defining related but clearly independent constructs of impulsive behavior. Criticized as being generally broad (Cyders, 2015), it is recommended the DSM-V term “impulsivity” be divided into separate constructs—one being lack of planning and regard for future consequences (choice impulsivity) and a separate one being a diminished ability to inhibit a natural, habitual or dominant “prepotent” response (rapid-response impulsivity).

Choice impulsivity in nicotine withdrawal research is highly dependent on trait impulsivity. Remarkably, both animal (Kolokotroni et al., 2014) and human (Harrison et al., 2009; Ashare and Hawk, 2012) studies show preference for smaller-sooner rewards during nicotine withdrawal but only for the low trait impulsivity groups. The mechanisms of this phenomenon yet have to be examined in laboratory settings, but unlike cocaine administration, which leaves a long-lasting negative impact on prolonged impulsive choice, in animal models, the effect of nicotine seems transient and normalizes in about 1 week (Kolokotroni et al., 2014). Smoking abstinence robustly reduces the ability to inhibit prepotent responding (Kozink et al., 2010) and administration of nicotine replacement results in the reversal poor rapid-response impulsivity (Larrison et al., 2004; Dawkins et al., 2007). The extent to which nicotine reverses rapid-response impulsivity predicts relapse during the first week of quitting smoking (Powell et al., 2004).

The overlapping effect of nicotine and insulin is evident in rapid-response impulsivity. Just as nicotine reverses the detrimental response, so does insulin. Insulin robustly and swiftly induces the cell surface expression and function of dopamine transporter in dopamine terminals (Patterson et al., 1998; Owens et al., 2005; Williams et al., 2007; Figlewicz and Benoit, 2008; Lute et al., 2008). Whereas, administration of insulin alone to nucleus accumbens decreases electrically evoked dopamine release, it enhances the release of dopamine when co-administered with cocaine due to insulin's effect on DAT function and enhancement of dopamine reuptake. Similar results were shown behaviorally. Rats receiving insulin demonstrated reduced rapid-response impulsivity, and co-administration of insulin and cocaine increased cocaine's induction of impulsive behavior (Schoffelmeer et al., 2011). The parallel cellular and behavioral findings of insulin's mechanism on the type of impulsivity particularly important in nicotine dependence identify insulin manipulation as a novel strategy for smoking cessation. Clinical studies need to be designed carefully, though, to evaluate whether intranasal insulin can be administered at the time of relapse. It is reasonable to hypothesize that co-administration of nicotine and insulin would worsen rapid response impulsivity more than either agent alone. If so, methods to address potential impeding of smoking cessation efforts would require a detailed evaluation.

## Mood

Anhedonia spikes within the first day of smoking cessation. It significantly predicts smoking cessation even after adjustment for related withdrawal symptoms such as, nicotine craving (Cook et al., 2015). Hence, any smoking cessation treatment addressing symptoms associated with discontinuation from smoking should, in theory, also act as an antidepressant. This is in line with the evidence that antidepressant buproprion is an effective agent for smoking cessation. The underlying cellular mechanisms and anatomical regions of withdrawal-induced anhedonia, though, are not well understood (Picciotto et al., 2008), though evidence suggests involvement of the β2 nicotinic acetylcoline receptor subunit (Stoker et al., 2015).

Insulin's role in mood modulation is in the beginning stage of investigation in both animal and clinical models. There is clear evidence of the involvement of insulin in anhedonia—the brain insulin receptor knockout NIRKO mice exhibit depressive behavior due to overactive monoamine oxidase activity and a subsequent increased dopamine turnover (Kleinridders et al., 2015). The hallmark of the phenotype is a mitochondrial dysfunction which is the cellular signature of insulin resistance. In addition, when a “next generation antidepressant” L-acetylcartinine (LAC) is administered to a genetic rat model of depression (Flinders Sensitive Line), the treatment reduces insulin and glucose levels, suggesting an insulin resistant state that responds to LAC. As such, the anhedonia of a large subset of smokers who are insulin resistant due to nicotine's well know insulin resistance-inducing actions in the periphery (Bajaj, 2012; Bergman et al., 2012), may benefit from targeted normalization of insulin signaling. In this case, treatments with insulin sensitizers would be warranted. Whether insulin co-administration would be of benefit, would need to be further evaluated.

Clinical administration of intranasal insulin for mood-related outcomes has resulted in mixed findings. Whereas, a study by Benedict et al. (2004) in healthy volunteers reported enhanced mood, a more recent study by Cha et al. (2017) did not find any changes in a cohort of major depressive disorder patients. Intranasal insulin 40 IU was administered four times per day in both studies. Compliance is a major limitation of these studies due to a multiple time per day drug regimen. Intranasal insulin studies with twice daily dosing have been executed and replicated successfully (Craft et al., 2012, 2017). Perhaps more importantly, intranasal insulin in all the published intranasal insulin studies thus far is compounded using the commercially available subcutaneous route form which causes severe nasal irritation and burning (Hamidovic et al., 2017). Hence, clinical investigators of intranasal insulin trials may have overlooked the issue of medication adherence assuming that, based on collecting returned medication, the medication was taken correctly. However, as discussed in Dresser (2013), the tendency of the research community to focus on the design and results while viewing adherence as “a nuisance and a somewhat tangential concern” possibly contributed to the mixed findings with intranasal insulin for its potential mood enhancing properties. In order to further study intranasal insulin in large scale clinical trials, insulin will have to be reformulated to be appropriate for nasal administration. Medication adherence may be monitored using newer methodologies such as, Artificial Intelligence platforms. Otherwise, adherence will be compromised with resulting inaccurate effect size estimates.

## Conclusion

Insulin receptors are widely distributed throughout the brain (Kleinridders et al., 2014) where their activation mediates synaptic plasticity and neurotransmitter signaling. As such, intranasal insulin is currently being evaluated for numerous psychiatric and neurologic conditions. At the time of this review, after excluding clinical trials of unknown status, clinicaltrials.gov registers 44 clinical studies with intranasal insulin. Having already shown that intranasal insulin is effective in decreasing nicotine cravings (Hamidovic et al., 2017), this review also evaluated whether additional mediators may be targeted by the treatment. Based on substantial evidence that disruptions in insulin signaling contribute to increased food intake, intranasal insulin administration may reduce weight gain during smoking cessation, which, if proven to be effective would have a substantial public health impact. Although animal models demonstrate the role of insulin in anhedonia, how this translates clinically is still unknown. Similar to the actions of psychostimulants, insulin induces the expression of dopamine transporter, thereby reducing rapid response impulsivity. This effect has yet to be shown in human behavioral models. Being a unique neuropeptide, which can be delivered centrally without peripheral absorption, insulin may have important effects on certain mediators of smoking persistence, thereby increasing positive smoking cessation outcomes.

## Author contributions

The author confirms being the sole contributor of this work and approved it for publication.

### Conflict of interest statement

The author declares that the research was conducted in the absence of any commercial or financial relationships that could be construed as a potential conflict of interest.
